# Exposure to Sub-inhibitory Concentrations of the Chemosensitizer 1-(1-Naphthylmethyl)-Piperazine Creates Membrane Destabilization in Multi-Drug Resistant *Klebsiella pneumoniae*

**DOI:** 10.3389/fmicb.2019.00092

**Published:** 2019-02-13

**Authors:** João Anes, Sathesh K. Sivasankaran, Dechamma M. Muthappa, Séamus Fanning, Shabarinath Srikumar

**Affiliations:** ^1^School of Public Health, Physiotherapy and Sports Science, Centre for Food Safety, Science Centre South, University College Dublin, Dublin, Ireland; ^2^Genome Informatics Facility, Iowa State University, Ames, IA, United States; ^3^Institute for Global Food Security, Queen’s University Belfast, Belfast, United Kingdom

**Keywords:** *Klebsiella pneumoniae*, chemosensitizers, 1-(1-naphthylmethyl)-piperazine, membrane destabilization, NMP, secondary effects

## Abstract

Antimicrobial efflux is one of the important mechanisms causing multi-drug resistance (MDR) in bacteria. Chemosensitizers like 1-(1-naphthylmethyl)-piperazine (NMP) can inhibit an efflux pump and therefore can overcome MDR. However, secondary effects of NMP other than efflux pump inhibition are rarely investigated. Here, using phenotypic assays, phenotypic microarray and transcriptomic assays we show that NMP creates membrane destabilization in MDR *Klebsiella pneumoniae* MGH 78578 strain. The NMP mediated membrane destabilization activity was measured using β-lactamase activity, membrane potential alteration studies, and transmission electron microscopy assays. Results from both β-lactamase and membrane potential alteration studies shows that both outer and inner membranes are destabilized in NMP exposed *K. pneumoniae* MGH 78578 cells. Phenotypic Microarray and RNA-seq were further used to elucidate the metabolic and transcriptional signals underpinning membrane destabilization. Membrane destabilization happens as early as 15 min post-NMP treatment. Our RNA-seq data shows that many genes involved in envelope stress response were differentially regulated in the NMP treated cells. Up-regulation of genes encoding the envelope stress response and repair systems show the distortion in membrane homeostasis during survival in an environment containing sub-inhibitory concentration of NMP. In addition, the *lsr* operon encoding the production of autoinducer-2 responsible for biofilm production was down-regulated resulting in reduced biofilm formation in NMP treated cells, a phenotype confirmed by crystal violet-based assays. We postulate that the early membrane disruption leads to destabilization of inner membrane potential, impairing ATP production and consequently resulting in efflux pump inhibition.

## Introduction

Bacterial multi-drug resistance (MDR) is a serious threat to infectious disease therapy (Van Boeckel et al., 2015). Drug resistance is now recognized by the World Health Organization as one of the most significant challenges of the 21^st^ century (World Health Organization, 2014). Currently, 25,000 people die each year in Europe alone due to multi-drug resistant (MDR) bacterial infections costing European Union € 1.5 billion annually (Davies et al., 2013). It is therefore imperative that new technologies be developed to mitigate drug resistance in bacteria.

The mechanisms used by bacteria to combat antimicrobial agents are (1) Reduced permeability of the antimicrobial compound; (2) Increased efflux of the molecule; (3) Gene mutation dependent modification of antimicrobial targets; and (4) Direct inactivation of drug molecules (Blair et al., 2015). The genes encoding proteins responsible for various antibiotic resistance mechanisms are frequently located on mobile genetic elements (MGE) such as plasmids, transposons, integrons and are disseminated *via* horizontal transfer (Carraro et al., 2014; Gillings, 2014).

Chromosomally encoded efflux pumps are membrane associated, ubiquitous in bacteria and act by extruding antimicrobial compounds from the bacteria – presenting a major challenge to antibiotic therapy (Anes et al., 2015; Blair et al., 2015). Antimicrobial concentration in the cytoplasm is thereby reduced leading to the development of resistance. Using an *in vitro* hollow-fiber infection model, it was shown that over-expression of efflux systems facilitates bacteria to initially adapt to antibiotic exposure, thereby acting as a primary mechanism of defense. This, in turn, provides time for the bacterium to evolve a mechanized framework of high-level resistance *via* target gene mutations (Singh et al., 2012). It is this initial tolerance in bacteria that precedes the fully expressed antimicrobial resistance phenotype as shown recently by *in vitro* evolution experiments (Levin-Reisman et al., 2017).

Five families of efflux pump transporters are recognized in bacteria, including those belonging to the RND (Resistance–Nodulation–Division) superfamily (Li et al., 2015). Structurally RND pumps are very complex with an internal membrane complex spanning inner membrane, periplasmic space, and outer membrane. This tripartite structure consists of an inner membrane efflux transporter with broad substrate specificity, outer membrane channel which extrudes the substrate across the outer membrane and the periplasmic adapter protein (Blair and Piddock, 2009; Nikaido and Pages, 2012). AcrAB-TolC is the most widely studied RND system in *E. coli* and it is often found overexpressed in MDR *E. coli* pathogens (Nikaido, 2011; Li et al., 2015). Resistance to various classes of antimicrobial agents have been associated with the overexpression of AcrB (Okusu et al., 1996; Li and Nikaido, 2009). The AcrAB-TolC pump has also been implicated in virulence and biofilm formation (Hirakata et al., 2009; Martinez et al., 2009; Baugh et al., 2014). RND pumps not only extrude antimicrobial compounds but also toxic cellular by-products (Okusu et al., 1996; Huguet et al., 2013; Ruiz and Levy, 2014) highlighting their important role in mediating bacterial stress responses and pathogenicity (Anes et al., 2015; Modarresi et al., 2015; Buckner et al., 2016).

The importance of efflux pumps in antimicrobial resistance has emphasized the utility of these pumps as primary targets for novel chemotherapeutic intervention strategies. Antimicrobial compounds when used in combination with efflux pump inhibitors (EPIs), or chemosensitizers, rendered drug resistant bacterial cells susceptible by facilitating a reduction in the minimum inhibitory concentration (MIC) of a given antimicrobial compound (Pages and Amaral, 2009; Song and Wu, 2016). In the past 20 years many chemosensitizers like PAβN, NMP, D13-9001, *etc.* have been developed (Li et al., 2015).

1-(1-Naphthylmethyl)-piperazine (NMP) is an arylpiperazine compound which is being investigated as a chemosensitizer. It was initially identified in a screen of N-heterocyclic organic compounds with activity against *E. coli* (Bohnert and Kern, 2005). Ethidium Bromide accumulation studies suggested that both AcrAB and AcrEF efflux pumps are inhibited by NMP in a dose dependent manner (Bohnert and Kern, 2005). NMP binds to the Phe residue rich lower pocket of AcrB binding site (Vargiu and Nikaido, 2012). Since this binding is weaker than normal AcrAB-TolC substrates, it was suggested by molecular docking experiments that NMP might act as a substrate of the AcrAB-TolC pump. Even though NMP acts as the substrate, during the process of pumping, NMP moves from the proximal to the distal pocket of AcrAB-TolC and instead of moving out they straddle the G-loop. This G-loop straddling interferes with normal substrate movement resulting in drug resistance reversal (Li et al., 2015).

1-(1-Naphthylmethyl)-piperazine was reported to reverse MDR in *Acinetobacter baumannii* (Pannek et al., 2006), *Vibrio cholerae* (Bina et al., 2009), *Staphylococcus aureus* (Li et al., 2011), *E. coli* (Kern et al., 2006), *Citrobacter freundii, Enterobacter aerogenes*, and *Klebsiella pneumoniae* (Schumacher et al., 2006). When used as an adjuvant, NMP reversed bacterial resistance against several compounds including dyes, phenicol-based drugs, fluoroquinolones, rifampicin, linezolid, tetracyclines, and others (Bohnert and Kern, 2005; Kern et al., 2006; Schumacher et al., 2006, 2007; Li et al., 2011; Marchetti et al., 2012). Furthermore, NMP was also reported to inhibit the secretion of two virulence factors, the cholera toxin and the toxin-co-regulated pilus in *Vibrio cholerae* (Bina et al., 2009). Molecular docking experiments reported that NMP binds to the distal pockets of the AcrB protein and competes with the antimicrobial agent for binding space within the efflux pump (Takatsuka et al., 2010; Vargiu and Nikaido, 2012; Schuster et al., 2014). Having greater affinity, NMP binds preferentially to the AcrB distal pocket than the antimicrobial agent (Poole and Lomovskaya, 2006) enabling the drug to concentrate in the bacterial cytoplasm and exert its bactericidal effect.

Phenotypically, efflux pump inhibition mediated by NMP is well-characterized (Takatsuka et al., 2010; Vargiu and Nikaido, 2012; Schuster et al., 2014). However, the secondary stress responses induced within bacteria following NMP exposure remain uncharacterized. Also, the cascade of phenotypic and genotypic events leading to efflux pump inhibition remains unknown. Taking cue from previous studies using PAβN (phenylalanine-arginine β naphthylamide) (Lamers et al., 2013; Misra et al., 2015) and PQQ4R -(4-(2-(piperazin-1-yl) ethoxy)-2-(4-propoxyphenyl) quinolone) (Machado et al., 2017) where efflux pump inhibition was preluded by widespread bacterial membrane destabilization, we hypothesized that the same could be true for NMP as well. To test this, we subjected NMP treated MDR *K. pneumoniae* MGH 78578 cells to a series of phenotypic assays testing bacterial membrane viability and found that NMP introduced widespread destabilization of the bacterial membrane. To further understand the genetic mechanisms underlying the membrane de-stabilization leading to efflux pump inhibition, we conducted RNA-seq on NMP treated bacterial cells to RNA-seq and transcriptional signals underlying the process were identified.

## Materials and Methods

### Chemical Compounds and Bacterial Isolates

Multi-drug resistance *K. pneumoniae* MGH 78578 (ATCC^TM^700721) is a clinical sputum isolate originally isolated in 1994 and was purchased from the American Type Culture Collection. This strain was selected for this assay mainly because it is a multi-drug resistant type strain (Ogawa et al., 2005). Recently we elucidated the resistance profile of this organism and identified that strain is resistant to at least 15 antibiotics (Anes et al., 2017). Moreover, the efflux pumps present in this strain is well-characterized (Li et al., 2008; Ogawa et al., 2012). The main advantage was that the whole genome sequence of this is available in NCBI (Refence Sequence NC_009648.1) which made it easier for us to map our RNA-seq data. This bacterium was grown in Müeller-Hinton (MHB) broth and agar (MHA) (Sigma, Dublin, Ireland). The EPI compound 1-(1-naphthylmethyl)-piperazine (NMP) (Sigma) was prepared at defined concentrations according to the manufacturers’ instructions.

### Membrane Destabilization Assay

1-(1-Naphthylmethyl)-piperazine mediated destabilization of the cell wall was determined according to published protocols (Misra et al., 2015) with minor modifications. Briefly *K. pneumoniae* MGH 78578 were grown at 37°C in MHA to 10^8^ bacterial cells/mL, washed twice, and resuspended in PBS. NMP (250 μg/mL) was added to washed bacterial cells resuspended in PBS containing nitrocefin (100 μg/mL). The assay was carried out in a 96-well plate using two technical replicates each with 100 μL. Absorbance was recorded at 492 nm every 5 min for a total of 100 min and results converted to nitrocefin hydrolysate produced (measured in μM). Polymyxin B (2 μg/mL) (corresponding to 2x minimum bactericidal concentration) was used as a positive control for membrane disruption. Hydrolysis rates were extracted from the exponential slope of the kinetic curves and reported as nitrocefin hydrolysate produced per minute. A standard curve was prepared from the complete hydrolysis of nitrocefin by resuspending 1 mL of an overnight culture of *K. pneumoniae* MGH 78578 at different concentrations of nitrocefin (3.125- to 100-μM) and incubated at 37°C for 20 min. This assay was performed in duplicate.

### Assaying Membrane Potential Measurements Using Flow Cytometry

The effect(s) of NMP on membrane integrity were evaluated using the BacLight Bacterial Membrane Potential kit (Thermo Fisher, Dublin, Ireland) following manufacturer’s instructions. First an overnight culture of *K. pneumoniae* MGH78578 in MHB was sub-cultured into fresh 50 mL MHB and grown at 37°C until OD_600nm_ 0.3 was reached. The culture was then diluted 10 times in PBS containing NMP at 125- and 250-μg/mL in the presence and absence of glucose (50 mM) at room temperature. Carbonyl cyanide m-chlorophenylhydrazone (CCCP) was also added to the cells diluted in PBS to a final concentration of 100- and 50-μM in the presence and absence of glucose to serve as a depolarized (positive) control. Following the addition of the testing compounds, 10 μL of 3 mM DiOC_2_(3) (3,30- diethyloxacarbocyanine iodide) was added to each sample and incubated at room temperature for 20 min. Samples were run in a BD Accuri C6 flow cytometer (BD, Oxford, England) using a 488 nm laser. Emission fluorescence was detected using the green fluorescence filter 533/30 and the red fluorescence filter 670LP.

Data obtained for all samples were analyzed by using the BD Accuri C6 software V. 1.0.264.21. Unstained control samples and NMP solutions were used to locate the bacterial population, and the compound signal in the forward scatter channel, respectively (Supplementary Figure S1). Bacterial cells devoid of compound and DiOC_2_(3) were used to select the cells in the forward and side scatter. Depolarized samples with CCCP were used to identify the bacterial cells with an altered membrane potential. These data were reanalyzed using the FCS Express 6 Plus beta research edition (De Novo software). All results were expressed as an average ratio of red fluorescence to green fluorescence of three biological replicates. One-way ANOVA was used to compare the results against the control sample, cells with DiOC_2_(3).

### Assaying NMP-Mediated Variations in *K. pneumoniae* Metabolism Using the Phenotypic Microarray

The effect of NMP on the metabolism of *K. pneumoniae* MGH 78578 was evaluated using the OmniLog (Biolog, Inc., Hayward, CA, United States) phenotypic microarray platform, testing 10 microplates named PM1 through PM10 except for PM5. All plates contained defined several carbon-, nitrogen-, sulfur- and phosphorous-substrates, ions, and osmolytes at different concentrations and pH (Bochner et al., 2001). *K. pneumoniae* MGH 78578 was grown at 37°C on MHA. Several colonies were picked with a sterile cotton swab and suspended in 15 mL physiological saline containing DMSO or NMP at 0.5% (v/v) and 250 μg/mL, respectively, until a cell density of 42% transmittance (T) was reached in a turbidimeter (Biolog, Inc., Hayward, CA, United States). Each 15 mL suspension was then added to 75 mL of physiological saline containing dye A, along with either DMSO or NMP at the concentrations mentioned previously. PM plates 1–2 were inoculated with the previous mixture. Plates PM 3–8 were inoculated with inoculating fluid-0 (IF-0) containing sodium pyruvate as carbon source. Finally plates PM 9–10 were inoculated with the inoculating fluid-10 (IF-10). One hundred microliters of each mixture were inoculated into each well of the microplates. All PM microplates were incubated at 37°C in an OmniLog reader and monitored for 72 h. Data was analyzed using DuctApe software v 0.17.4 (Galardini et al., 2014). Each isolate was analyzed in duplicate (Supplementary Table S1; WS 1).

### Bacterial Growth Curves in the Presence of NMP

The effects of NMP on the growth of *K. pneumoniae* MGH 78578 was carried out in MHB in 250 mL conical flasks with 200 rpm at 37°C. Bacterial cells were inoculated at OD _600__nm_ 0.005 and allowed to grow until OD _600__nm_ 0.6. Here NMP and DMSO were added at 250 μg/mL and 0.5% (v/v) respectively. Bacterial growth was enumerated followed by serial dilution and plating onto MHA. Samples were further taken for RNAseq and TEM imaging at time points 15 min, 3 and 21 h post-NMP exposure.

### RNA Purification, Sequencing (RNA-seq), Read Mapping, Computational Analysis, and Quantitative Real-Time PCR

RNA was isolated from NMP treated *K. pneumoniae* MGH 78578 using RNAeasy extraction kit (Qiagen, Hilden, Germany) following manufacture’s instructions. Samples of the bacterial culture were taken at 15 min and 21 h post-NMP exposure and were then combined with two volumes of RNAprotect (Qiagen, Hilden, Germany). Bacterial cultures treated with DMSO for the same two-time points served as the negative control. The NMP/DMSO treatments and RNA isolations were carried out in biological replicates amounting to a total of eight libraries. The isolated RNA was then treated with Turbo DNase (Ambion, Foster City, CA, United States) to remove any contaminating DNA. DNAse treated RNA samples were subject to integrity testing and quantification using both the Bioanalyzer 2100 RNA 6000 nanochip (Agilent Technologies, Santa Clara, CA, United States) and the ND-1000 (Nanodrop Technologies, Wilmington, DE, United States), respectively. Purified RNA samples were then preserved at −80°C.

The library preparation and subsequent sequencing were conducted commercially at the Eurofins Genomics (Ebersberg, Germany). Ribo-Zero rRNA removal Kit for Bacteria (Illumina, San Diego, CA, United States) was used to carry out the depletion of ribosomal DNA according to the manufacturer’s instructions. Libraries were created using NEBNext^®^ Ultra^TM^ Directional RNA Library Prep Kit (New England BioLabs, Frankfurt, Germany). Pooled libraries were loaded on the cBot (Illumina, San Diego, CA, United States) and cluster generation was performed according to manufacturer’s instructions. Single-end sequencing using 125 bp read length was performed on a HiSeq2500 machine (HiSeq Control Software 2.2.38) using HiSeq Flow Cell v4 and TruSeq SBS Kit v4 (Illumina). Raw sequencing read data was processed using RTA version 1.18.61 and CASAVA 1.8.4 to generate FASTQ-files. Genomic cDNA libraries were prepared using the TruSeq Stranded Total RNA Library Prep Kit (Illumina, San Diego, CA, United States) with Ribo-Zero to deplete ribosomal RNA (rRNA). An average of 1.48 Gb of raw sequence data was obtained per sample, in 125 bp single end reads.

#### Mapping of Sequenced Reads

Sequence reads were mapped against the *K*. *pneumoniae* MGH 78578 (NC_009648) reference genome using Segemehl (Hoffmann et al., 2009) with an accuracy set to 100%. To increase mapping quality, unmapped reads were sequentially truncated by one nucleotide beginning from the 3′-end of each read and then re-mapped. This process was iterated until a given read mapped to a single location on the bacterial chromosome, or a minimal read length of 20 nucleotides was reached. Only uniquely mapped reads were used for downstream computational analysis (Supplementary Table S1; WS 2).

#### Computational Analysis of RNA-seq Data

Computational analysis of RNA-seq data was performed using R (version 3.2.4^1^). To calculate the expression level of genes, the raw read counts were normalized using the *VOOM* function (Law et al., 2014) in the *limma* package (Ritchie et al., 2015). More specifically, counts were converted to log_2_ counts per mllion (log_2_ CPM), quantile normalized, and precision weighted using the *VOOM* function. A linear model was then fitted to each gene, empirical Bayes moderated *t*-statistics and its corresponding *P-values* were used to assess differences in expression (Smyth, 2004; Law et al., 2014). To account for multiple comparisons, Benjamini–Hochberg corrected *P-values* were computed. As reads for duplicated coding genes (paralogs) or duplicated small RNAs cannot be mapped unequivocally, these genes appear in the analysis as unmapped. The sequence reads can be visualized in Integrated Genome Browser (version 9.0.0) (Nicol et al., 2009). The read depth was adjusted in relation to the cDNA library with the lowest number of reads (Skinner et al., 2009).

#### GEO Accession Number

The RNA-seq data generated from this study are deposited in NCBI-GEO and are available under the accession number GSE122651.

#### Quantitative Reverse Transcriptase PCR

Quantitative Reverse Transcriptase PCR (qRT-PCR) experiments were performed to validate the RNA-seq data. RNA purified from the time points described previously was converted into cDNA using the High-Capacity RNA-to-cDNA Kit (Thermo Fisher, Dublin, Ireland). Target genes were designed with IDT PrimeTime qPCR assays that included 6-FAM/ZEN/IBFQ double-quenched probes. Primers (Supplementary Table S1; WS 4) were synthesized by Integrated DNA Technologies (IDT, Leuven, Belgium). qRT-PCR was performed using the synthesized cDNA along with the PrimeTime qPCR assays using the PrimeTime Gene Expression Master Mix in an Eppendorf Mastercycler realplex *ep gradient S* (Eppendorf, Arlington, United Kingdom) according to the manufacturer’s instructions. Samples were run for 3 biological replicates each with three technical replicates. Data were analyzed using realplex software. The relative fold-increases in expression levels (ΔC_t_) were normalized based on the gene expression levels of the housekeeping gene *rpoB.* Comparative quantification was carried out using the ΔΔC_t_ approach in the control (DMSO) *versus* the comparator (NMP) samples.

### Transmission Electron Microscopy (TEM)

*Klebsiella pneumoniae* MGH 78578 was observed in the TEM in the presence of NMP (250 μg/mL) and DMSO 5% (v/v) following incubation in MHB at 37°C. NMP/DMSO treated bacterial cells were harvested at the corresponding time points described previously, centrifuged (at 13,000 rpm, 5 min, 4°C) and washed three times with cold PBS. Samples were fixed in PBS with 2.5% (v/v) glutaraldehyde and incubated overnight at 4°C. The fixed cells were treated with Sørensen’s phosphate buffer and 1% (w/v) osmium tetroxide (TAAB Laboratories Equipment, Aldermaston, United Kingdom) for 60 min at room temperature, followed by a washing step with Sørensen buffer. Cells were then centrifuged, and the pellets recovered were dehydrated in ethanol solutions of increasing concentrations and embedded in propylene oxide for 30 min. A mixture of propylene oxide – epoxy resin (Epon 812; Tousimis Research, Corp., Rockville, MD, United States) at a ratio of 1:1 was added to the pelleted cells and incubated at room temperature for 1 h. The epoxy resin was then embedded into the pellet, which was polymerized by incubation for 2 h at 37°C. After this step, cells were subjected to centrifugation and the pellet was re-suspended in epoxy resin which was polymerized by incubation at 60°C overnight. Ultrathin sections were collected on copper grids and stained with uranyl acetate and lead citrate. Observations were made using a FEI Tecnai transmission electron microscope (Tecnai, Co., Huston, TX, United States) operating at 120 kV. Electron microscope observations were documented through the acquisition of representative microphotographs for each time point.

### Inhibition of Biofilm Formation Using NMP

The effect of NMP on the efficiency of *K. pneumoniae* MGH 78578 to develop a biofilm was tested by growing the bacterial cells in M9 minimal media (Neidhardt et al., 1974) with NMP. Briefly, an overnight culture of *K. pneumoniae* MGH 78578 was sub-cultured and grown until the culture reached an OD_600__nm_ 0.3 in M9 minimal media (NH_4_Cl [1.9 mM], Na_2_HPO_4_ [42.3 mM], KH_2_PO_4_ [22 mM], NaCl [8.56 mM], MgSO_4_ [2 mM], CaCl_2_ [0.1 mM], and glucose [0.1% w/v]) in the presence and absence of NMP (250 μg/mL). Diluted cells were dispensed (as 200 μL volumes) across a 96-well microtiter plate and incubated statically for 24 h at 37°C.

Crystal violet (Sigma) was applied to stain the total biomass formed (Stepanović et al., 2007). Bacterial cells were washed with PBS and stained with crystal violet 0.4% (w/v) and incubated at room temperature for 15 min. After that period, cells were washed once more and fixed with 200 μL 33% [v/v] acetic acid. Absorbance was recorded in a Multiskan^TM^ FC Microplate Photometer (Thermo Fisher Scientific, Dublin, Ireland) at 570 nm. This assay was repeated three times for each isolate and each assay was carried out over 10 technical replicates. Results were compared against the control well-containing fresh M9 minimal media alone. Biofilm data was processed individually for each condition and comparisons between conditions were performed using Prism 6 (GraphPad) using Student’s *t*-test analysis. *P-values* of < 0.05 were considered significant.

**FIGURE 1:**
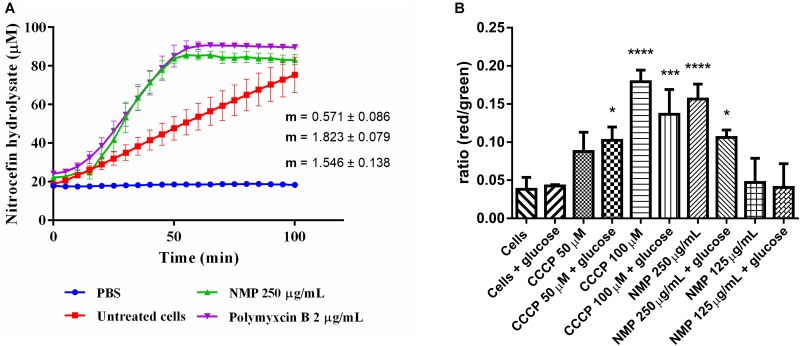
**(A)** Measurements of β-lactamase enzymatic activity in *Klebsiella pneumoniae* MGH 78578, in the presence of 250 μg/mL NMP. Activity was measured over 100 min at 37°C. Slope values of the activity curves represent the rate of nitrocefin hydrolysis/min. Polymyxin B was used as an internal control for cell wall disruption at a concentration of 2 μg/mL. Background absorbance values for nitrocefin, nitrocefin with NMP and nitrocefin with polymyxin B were subtracted from the corresponding samples. Slopes and standard deviations were calculated from three individual experiments with two technical replicates each. **(B)** Effect of NMP on the membrane potential of *K. pneumoniae* MGH 78578. Bacterial membrane potential was measured by flow cytometry using the Baclight kit (Invitrogen). Bacterial cells were diluted in PBS (control) and in PBS containing defined concentrations of the proton-motive force inhibitor CCCP and NMP in presence and absence of glucose. DiOC_2_(3) was added to all samples and fluorescence emission data collected in the green and red channels. Variations in the membrane potential cause a change in fluorescence of DiOC_2_(3) from green to red. Results were compared to the control sample [Cells + DiOC_2_(3)] by one-way ANOVA statistical test – ^∗^*P* < 0.05, ^∗∗∗^*P* < 0.001, ^∗∗∗∗^*P* < 0.0001.

## Results and Discussion

### Effect(s) of NMP in the Membrane Destabilization

Since chemosensitizers like PQQ4R and PAβN were observed to have a membrane destabilization effect in several bacteria (Lamers et al., 2013; Misra et al., 2015; Machado et al., 2017), our first hypothesis was that NMP might have similar effects on *K. pneumoniae* MGH78578. Previously we had determined the MIC of NMP on multi-drug resistant *K. pneumoniae* MGH78578 as 500 μg/mL (Anes et al., 2018). Here, we subjected *K. pneumoniae* MGH78578 to a β-lactamase assay in the presence of 250 μg/mL (0.5x MIC), defined as a sub-inhibitory concentration of NMP. β-Lactamase is a periplasmic enzyme, its extracellular release correlates with disruption of the outer membrane (OM) which can be assayed by the hydrolysis of the chromogenic substrate nitrocefin (O’Callaghan et al., 1972). Polymyxin B, an antibiotic, known to disrupt the bacterial membrane (Storm et al., 1977), was used as the positive control and untreated *K. pneumoniae* MGH78578 cells acted as the negative control. NMP treated cells were determined to have a β-lactamase activity of 1.8 μM hydrolyzed nitrocefin/min, similar to that of polymyxin B (1.5 μM/min) (Figure 1A). This assay showed that the OM of *K. pneumoniae* MGH78578 was disrupted following treatment with sub-inhibitory concentrations of NMP.

Any agent that damages the cell membrane should alter membrane permeability (MP). Therefore, to further confirm the NMP-mediated disruption of the bacterial cell membrane we assayed the fluctuations in the MP during NMP treatment. Here, we used the Baclight kit (Invitrogen) to assay the MP of NMP treated *K. pneumoniae* MGH 78578 (Spindler et al., 2011). This kit measures the accumulation of the fluorescent dye DiO_2_C(3) (3,30- diethyloxacarbocyanine iodide) within a cell with intact cell membrane measured as a shift from green to red fluorescence. The DiO_2_C(3) accumulation within a cell with depolarized membrane is indicated by a shift in fluorescence from red to green (Spindler et al., 2011). The uncoupling agent CCCP which is known to depolarize the bacterial membrane was used as the positive control at concentrations of 50 and 100 μM.

To standardize the protocol, bacterial autofluorescence was first checked by gating unstained bacteria, followed by the gating of the bacterial population using side scatter *versus* forward scatter (Supplementary Figure S1). Next NMP was gated to ensure that the background signal noise from the compound did not influence the emission readings being taken. Subsequently DiOC_2_(3)-, NMP-treated and untreated *K. pneumoniae* MGH78578 were assayed for their membrane potential using the 488 nm blue light laser (Texas Red filter – LP 670 nm and Green filter – BP 530/30 nm). Treatment with NMP reduced the membrane potential to levels similar to CCCP treated cells and significantly different from untreated cells (Figure 1B). Since membrane potential is associated with inner membrane, the DiO_2_C(3) accumulation assay indicates that inner membrane is destabilized. However, the effect of NMP on membrane potential was concentration dependent. While at 250 μg/mL (0.5 MIC) there was a significant reduction in bacterial membrane potential, at 125 μg/mL (0.25 MIC) the reduction was negligible. Stabilization of membrane potential was noted when NMP treated cells were grown in the presence of glucose confirming an earlier observation that glucose restores membrane potential by activating ATP dependent mechanisms (Paixao et al., 2009). Results from β-lactamase and DiO_2_C(3) accumulation assays indicate that both outer and inner membranes of *K. pneumoniae* MGH 78578 cells are destabilized during NMP exposure.

**FIGURE 2:**
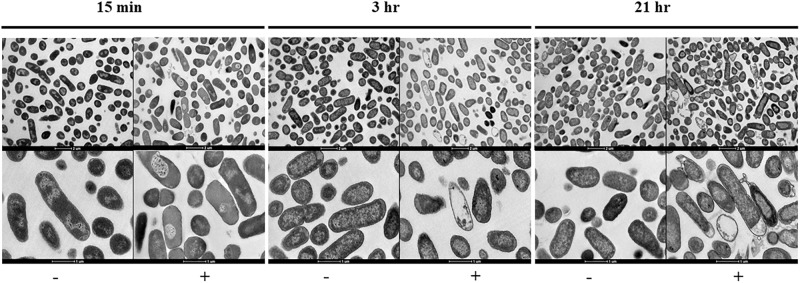
Transmission electron micrographs sowing the morphological changes observed on NMP treated *K. pneumoniae* MGH 78578. Images were collected after 15 min, 3- and 21-h of exposure to NMP. The images in the top panel were magnified 6,000X, bottom panel images shown were magnified 16,500X. The sign ‘–’ denotes DMSO and ‘+’ denotes DMSO plus NMP.

To visually interpret the effect of NMP on *K. pneumoniae* MGH 78578, we carried out transmission electron microscopy (TEM) on NMP and DMSO-only treated cells. *K. pneumoniae* MGH 78578 was exposed to 250 μg/mL NMP at three different time points – 15 min, 3- and 21-h. TEM images confirmed that for NMP treated *K. pneumoniae* MGH 78578, these cells underwent discernible morphological changes in their bacterial membrane (Figure 2). Cytoplasmic condensation and cell content leakage were noted following exposure to NMP as early as 15 min. Prolonged exposure exacerbated the above effects with more evident loss of cell content and coagulation of cytoplasmic material observed post 21 h NMP treatment. All these observations confirmed that NMP specifically affected cell membrane integrity.

### NMP Treatment Affected *K. pneumoniae* Metabolism

Biochemical composition of the bacterial membrane lipids is dependent on cell growth conditions and subsequent metabolic variations (Sohlenkamp and Geiger, 2016). To understand the metabolic variations in NMP treated *K. pneumoniae* MGH 78578 compared to untreated cells, we assessed the ability of NMP treated cells to metabolize several substrates using a phenotypic microarray (Biolog). This assay measures the respiration of bacterial cells, by detecting colorimetric changes in the presence of a tetrazolium dye. Since DMSO is the solvent used for NMP, DMSO treated cells were used as control to elicit any possible metabolic responses.

**FIGURE 3:**
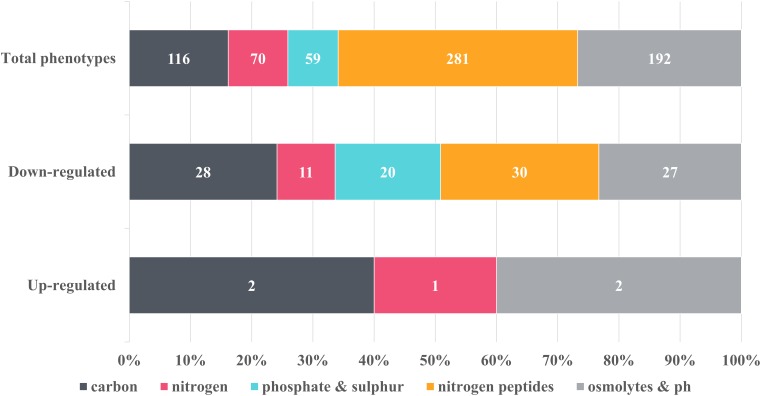
Overview of the metabolic changes detected in NMP-treated *K. pneumoniae* MGH 78578 as observed in the phenotypic microarray platform. Phenotypes were grouped into classes and the number of representatives contained within each class was indicated on the graph. Control phenotypes were assessed in the presence of DMSO, the solvent used to solubilize NMP. Raw data was analyzed using DuctApe software (v 0.17.4) and significant changes were considered for Δ activities lower and higher than –2 and 2 respectively. Five metabolic pathways were considered to be up-regulated in the presence of 250 μg/mL NMP with 116 being down-regulated.

Of the total 597 substrates measured, NMP treated *K. pneumoniae* MGH 78578 showed decreased respiration on 116 substrates and increased respiration on five substrates (Figure 3). Increased respiration was noted on substrates like glycerol and D-tagatose, suggesting that NMP stressed cells improved glycerol uptake similar to *Saccharomyces cerevisae* during hyperosmotic stress (Petelenz-Kurdziel et al., 2013). NMP treated *K. pneumoniae* MGH 78578 also exhibited an increase in respiration rate in sodium nitrate. Since sodium nitrate is widely used as an electron acceptor during anaerobic respiration (Stewart and Cali, 1990), increased respiration rate suggests that NMP treated cells can endure anaerobic or microaerophilic conditions better than DMSO treated control cells. Of the 116 substrates on which NMP treated *K. pneumoniae* MGH 78578 exhibited a decreased respiration rate, the most significant was alkaline pH. The piperazine ring of NMP has a pKa of 9.73. So, at alkaline pH the ring will be protonated on the nitrogen positions (Wishart et al., 2018) making the compound highly toxic to the bacterial cells thereby reducing the respiration rate. Overall these data showed that *K. pneumoniae* MGH 78578 could adapt to metabolic pathways that contributed to the bacterial recovery during NMP stress.

**FIGURE 4:**
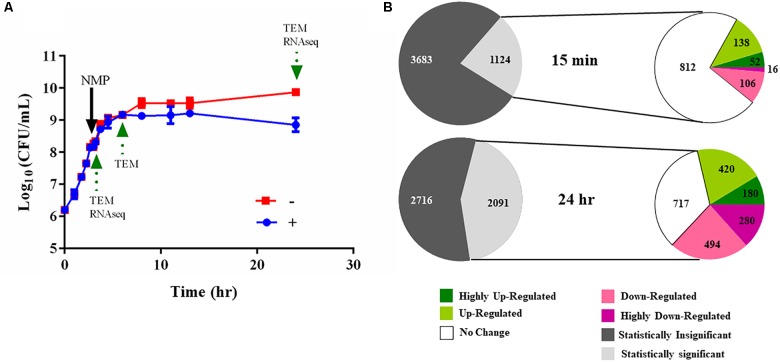
Experimental design of the RNA-seq experiments. **(A)** Typical bacterial growth curve measured at 37°C in MH broth in the presence of the 250 μg/mL NMP and the control solvent DMSO. NMP was added when OD_600__nm_ of the culture reached mid-log phase (0.6 approximately after 3 h of growth). The sign ‘–’ denotes DMSO and ‘+’ denotes DMSO plus NMP. Samples were taken for transmission electron microscopy (TEM) and for RNA purification as indicated by green arrows at time points 15 min, 3 and 21 h following exposure. **(B)** Overview of the number of the differentially regulated genes identified using RNA-seq. The gray and white colored pie chart [upper] depicts the number of genes for which statistically significant data (*Voom, P* < 0.05) was obtained from the two time points. The second pie chart [lower] depicts the number of up- and down-regulated genes at the respective time point. The color of each pie represents the relative gene expression based on the color scale given below.

### The Transcriptional Architecture of NMP Treated *K. pneumoniae* MGH 78578

We used RNA-seq to elucidate the transcriptional landscape of NMP stressed *K. pneumoniae* MGH 78578. Here, total RNA was isolated from bacterial cells grown in the presence of NMP (250 μg/mL) or DMSO (5%) for 15 min and 21 h (Figure 4A). The 15-min time point was used to simulate an *NMP shock* condition to identify early transcriptional signals following NMP exposure while the 21-h assay was selected to identify the adaptive responses to prolonged exposure – *NMP adaptive response*. About 62 million reads mapped uniquely across eight libraries making an average of 7.75 million reads/library, sufficient for robust transcriptomic analysis (Haas et al., 2012). The expression levels and differential regulation of the 4,809 *K. pneumoniae* MGH 78578 genes during NMP stress were calculated using the *Voom* approach (limma package). To confirm the reproducibility of the RNA-seq data, the correlation coefficients (Spearman) were calculated for normalized read counts and between two biological replicates, correlation co-efficient was ∼0.99 (Supplementary Figure S2), indicating significant statistical correlation between replicates. Statistically significant gene expression data was obtained for 1,124 genes at 15 min post-NMP exposure (*NMP shock*) and 2,091 genes at 21 h post-exposure (*NMP adaptive response*) (Figure 4B). Of those genes detected, during the *NMP shock*, 190 genes were up-regulated and 122 were down-regulated (>2 fold). During the *NMP adaptive response*, 600 genes were up-regulated while 774 genes were down-regulated.

**FIGURE 5:**
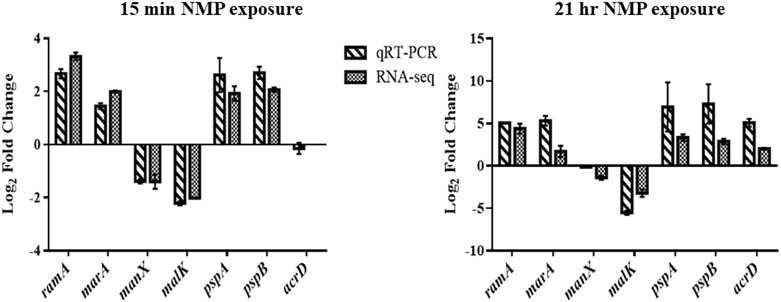
A figure showing the validation measurements taken comparing RNA-seq data and qRT-PCR.

AcrAB-TolC is an important RND efflux system in all Gram-negative bacteria (Li et al., 2015). Genes *acrAB* and *tolC* were up-regulated (threefold) (Supplementary Table S1; WS 3) in NMP stressed *K. pneumoniae* MGH78578 as observed earlier in *Salmonella* Typhimurium (Lawler et al., 2013). Similarly, the genes *marAB* (AraC family transcription regulator operon) were up-regulated 3.5 fold and *ramA* (a transcription regulator protein) had a 9.95 fold up-regulation in NMP exposed *K. pneumoniae*. This NMP mediated up-regulation of genes agreed with the expression patterns previously reported in the literature confirming the robustness of our analysis. qRT-PCR was conducted on representative differentially regulated genes to validate our RNA-seq based transcriptomic data. For all tested genes, the qRT-PCR data reflected the observations from our RNA-seq data further supporting the robustness of our RNA-seq based transcriptomic analysis (Figure 5).

Important examples of up-regulated genes identified during both *NMP shock* and *NMP adaptive responses* were *nhaA*, *degP*, *ramA*, *spy*, *pspABCD*, *pmrHFIK, mgtE*, etc. – showing bacterial response to maintain homeostasis during NMP stress. For example, NhaA is an integral membrane protein which is crucial for the maintenance of cytosolic pH and Na^+^ concentrations in *S.* Typhimurium (Lentes et al., 2014). The up-regulation of *mgtE* is to maintain Mg^2+^ homeostasis as reported earlier (Hattori et al., 2009). Lipid A modification plays a key role in the resistance to factors that compromise bacterial cell membrane (Moreira et al., 2013). Many lipid A modification genes were up-regulated in NMP stressed *K. pneumoniae* MGH 78578. The up-regulation of *eptB* [Kdo(2)-lipid A phosphoethanolamine 7-transferase which adds phosphatidylethanolamine to the outer Kdo residue of Lipid A], *lpxO* (α-keto glutarate dependent dioxygenase enzyme that hydroxylates the secondary acyl chain of Kdo2-lipid A in the presence of O_2_), *pmrHFIK* (biosynthesis of L-Ara4N of Lipid A) (Raetz et al., 2007) *etc.*, are responsible for an overall reduction in the negative charge on lipid A. Exogenic stress factors like heat shock, envelope stress, *etc.* generally result in the accumulation of toxic misfolded proteins in the bacterial periplasm, as observed in *S.* Typhimurium when exposed to ethanol and polymyxin B – induced the expression of the *spy* gene encoded chaperonic spheroplast protein y (Spy) (Jeong et al., 2017). In NMP stressed *K. pneumoniae* MGH 78578, continuous up-regulation of *spy* was noted in both the *NMP shock* and *NMP adaptive response* datasets. A similar up-regulation was noted for *degP*, chaperon degrading heat stress associated misfolded periplasmic proteins during heat stress, as reported previously in *E. coli* (Kim and Sauer, 2014). NMP exposed *K. pneumoniae* MGH 78578 also up-regulated periplasmic peptidylprolyl *cis–trans* isomerases which catalyzes the isomerization of peptidyl-prolyl bonds, a rate limiting step during protein synthesis (Obi et al., 2011). The up-regulation of *cpxP* shows that the protein represses CpxA inactivating the conjugative pilus system as seen in envelope stressed *E. coli* (Weatherspoon-Griffin et al., 2014). The phage shock protein operon (*pspABCD* and *pspG*) encoding responsive switch (Flores-Kim and Darwin, 2016) during membrane stress was also up-regulated during NMP exposure. All these transcriptomic responses show continuous efforts by the bacterium to maintain membrane homeostasis during NMP stress.

**FIGURE 6:**
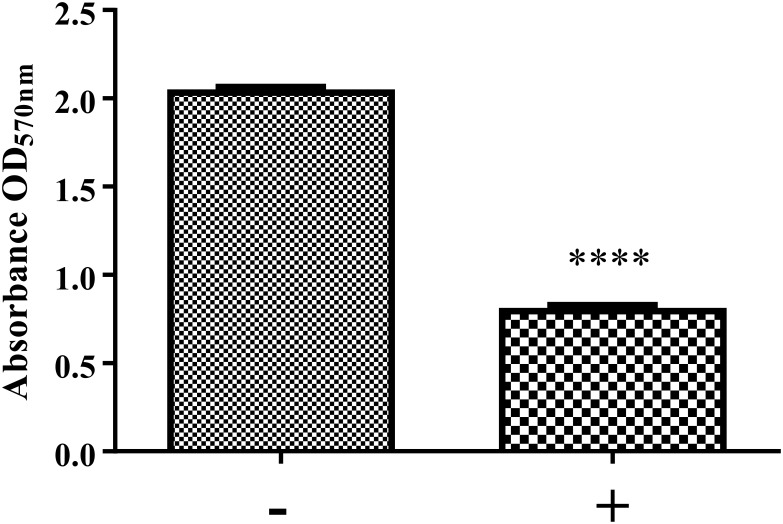
Biofilm formation of *K. pneumoniae* MGH 78578 in the presence and absence of NMP. Bacterial cells were grown statically in M9 minimal media in the presence of NMP at 250 μg/mL for 24 h at 37°C. The sign ‘–’ denotes DMSO and ‘+’ denotes DMSO plus NMP. Biofilm formation was measured by crystal violet method and compared between NMP treated and untreated cultures (^∗∗∗∗^*P* < 0.05).

**FIGURE 7:**
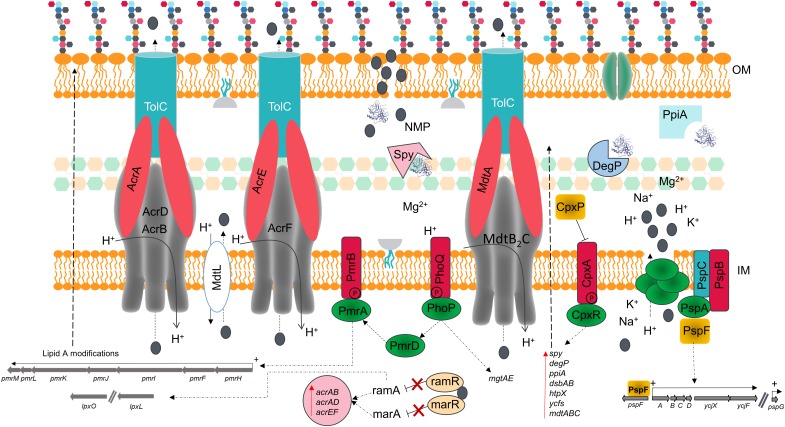
Schematic model summarizing the effects of NMP of the membrane of *K. pneumoniae* MGH 78578. The chemosensitizer NMP (indicated by the gray colored circle) crosses the cell membrane by disrupting the integrity of the lipid bilayer [both outer membrane (OM) and inner membrane (IM)]. This disruption causes the leakage of essential ions and cell content components leading to several structural and metabolic changes in a manner reminiscent of the action of similar cationic antimicrobial peptides.

Some genes were differentially regulated only during adaptive response. Important examples include *rpoH* (encodes σ^32^), *phoPQ* (two- component regulatory system) and *pmrD*. The up-regulation of *rpoH* had been noted in membrane stress associated *K. pneumoniae* (Ramos et al., 2016) and temperature stressed *E. coli* (Nonaka et al., 2006). Similarly, *phoPQ* was reported to be up-regulated when RNA-seq was used to study the transcriptional response of *K. pneumoniae* during exposure to colistin (Wright et al., 2015). A PhoP dependent gene *pmrD* [promoting lipid A modifications increasing bacterial resistance to cationic antimicrobial peptides (Rubin et al., 2015)] was also up-regulated 4.5 fold during adaptive NMP response. Genes encoding chaperone DnaK and co-chaperone DnaJ are also highly up-regulated (7 to 8 fold) during adaptive response to NMP. DnaK is known to directly interact with lipids and forms dimers with the membrane bilayer (Lopez et al., 2016). Moreover, when transcriptional response of a double deletion of *dnaKJ* was studied on a microarray, the expression of many inner membrane proteins were down-regulated (Fan et al., 2016). Overall many genes involved in the maintenance of the normal cell membrane were up-regulated during NMP exposure, a feature which shows that NMP mediated cell membrane destabilization causes the expected bacterial response, to maintain membrane homeostasis.

### The Inhibition of Bacterial Efflux in NMP Treated *K. pneumoniae*

1-(1-Naphthylmethyl)-piperazine is well-known as an EPI (Kern et al., 2006; Schumacher et al., 2006; Schuster et al., 2014). However, NMP treated *K. pneumoniae* MGH 78578 significantly up-regulated the expression of two major AraC-transcriptional global regulator genes, *ramA* and *marA.* NMP-mediated overexpression of *ramA* was reported previously in *S.* Typhimurium using promoter-*gfp* fusion experiments (Lawler et al., 2013). Molecular dynamics simulation experiments show that NMP functions as a substrate for the AcrAB-TolC pump and during pumping NMP straddles the G-loop, effectively blocking the efflux pump (Li et al., 2015). This leads to increased accumulation of the antibiotic in the bacterial cytoplasm reversing the resistance in multi-drug resistant bacteria (Vargiu and Nikaido, 2012). Global regulators like RamR can bind to some substrates of the AcrAB-TolC pump (Yamasaki et al., 2013). We postulate that NMP could bind to RamR, reducing its DNA binding efficiency subsequently increasing the expression of *ramA.* However, our postulation that NMP could bind RamR is hypothetical and will need further experiments for confirmation.

### The Effect of NMP Treatment in Biofilm Formation

Biofilm associated cells exhibit significantly higher tolerance/resistance to antibiotics (Hoiby et al., 2010). Increased expression of efflux pumps is one of the mechanisms that confer increased antibiotic resistance to biofilm associated cells (Gilbert et al., 2002). Therefore inactivation of efflux pumps is seen as a strategy to eradicate biofilm formation in bacteria (Baugh et al., 2014). Gene deletion based inactivation of efflux pumps showed inhibition of biofilm formation in *S.* Typhimurium (Baugh et al., 2014). Addition of EPIs also reduced biofilm formation but the rate of reduction varied with the type of EPI used. While Thoridazine and PAβN did significantly reduce the biofilm formation in *Staphylococcus aureus* and *Pseudomonas putida*, application of NMP did not produce significant reduction in biofilm formation in both these pathogens (Kvist et al., 2008). Interestingly, application of PAβN reduced the MBEC (minimum biofilm eradication concentration) of Ceftazidime and Doxycycline against *Burkholderia pseudomallei* biofilms (Sirijant et al., 2016), indicating compromised biofilm formation capability in PAβN treated bacterial cells.

The preparation and maintenance of biofilms is mainly coordinated by quorum sensing (QS), where signaling molecules like autoinducers (AIs) coordinate various bacterial behaviors in relation to bacterial cell density. Our RNA-seq data showed that the *lsr* operon encoding autoinducer-2 (AI-2) (Vendeville et al., 2005; Brito et al., 2013) was down-regulated during exposure to NMP (Supplementary Table S1; WS 3). This led us to hypothesize that the down-regulation of the *lsr* operon might reduce the production of AI-2, leading to a consequent reduction in the biofilm formation ability of NMP treated *K. pneumoniae* MGH78578. Our crystal violet staining analysis showed that treatment with NMP reduced the ability to form biofilms by 60% compared to untreated cells (Figure 6). When PQQ4R was used as the EPI, membrane destabilization along with impairment in the production of intracellular ATP was recorded, ultimately leading to the inhibition of efflux pump (Machado et al., 2017). In this case too membrane destabilization coupled with efflux pump inhibition could result in the inhibition of biofilm formation in NMP treated *K. pneumoniae* MGH78578.

## Conclusion

The alarming increase in antimicrobial resistance has driven the need for alternative approaches to control the spread of MDR bacteria. The use of chemosensitizers like NMP may contribute to the development of a new antimicrobial therapy, by allowing the use of older antimicrobial drugs in combination with NMP-like drugs, thereby increasing their efficacy. Even though efflux pump inhibition is the main theme of NMP action, we propose that membrane destabilization is an additional effect of NMP on bacterial cells. We confirm this using β-lactamase, membrane potential fluctuation and TEM assays in *K. pneumoniae* MGH78578. This effect is observable as early as 15 min post-NMP treatment. Our investigations using phenotypic microarray and RNA-seq revealed that cells actively synthesize proteins required to repair and maintain membrane homeostasis. This disruption of the bacterial membrane could lead to the loss of intracellular content changing the proton gradient, confirmed by the changes in the membrane potential seen in the experiments and by microscopy. *acrB* is found up-regulated in the presence of NMP and NMP is found to bind to the distal pocket of AcrB (Schuster et al., 2014; Vargiu et al., 2014). The up-regulation of other efflux systems such as AcrD, AcrF, and MdtB_2_C could be an alternative to the blocked AcrB efflux pump. The loss of membrane homeostasis caused the activation of PhoPQ which in turn activates PmrD, a regulator of the PmrAB two-component system (Cheng et al., 2010; Rubin et al., 2015). Activation of the PmrAB system induces the up-regulation of several LPS-based modification genes *via* the operon *pmrMLKIFH* (Farizano et al., 2012). Induction of RamA can in turn induce different LPS modification enzymes (Schneiders et al., 2003). These modifications are expected to set the bacterial membrane net charge toward a predominantly positive charge and prevent further interaction of NMP with the cell wall (Figure 7). This paper therefore provides unique insights in to the effects of NMP on a bacterial cell.

## Author Contributions

JA, SF, and SS designed the experiments. JA performed the experiments. JA and SS analyzed the data. DMM performed the qRT-PCR. SKS performed the RNA-seq analysis. JA, SF, and SS wrote and revised the manuscript into which DMM, SKS commented.

## Conflict of Interest Statement

The authors declare that the research was conducted in the absence of any commercial or financial relationships that could be construed as a potential conflict of interest. The reviewer ZS declared a shared affiliation, with no collaboration, with one of the authors, SKS, to the handling Editor at the time of the review.

## Footnotes

1 https://www.r-project.org/

## Supplementary Material

FIGURE S1 Sequential gating strategy applied to characterize *K. pneumoniae* MGH 78578 membrane potential shifts in the presence of the chemosensitizer NMP. The bacterial population was first identified with an unstained sample to check for autofluorescence using 633 nm laser with the filter 675/25 nm; gated samples (singlets 1) were next analyzed based on the forward scatter and gated as singlets 2. Finally, bacterial population was gated using the side scatter *versus* forward scatter. In order to ensure that the signal from the compounds did not affect the gating, a sample of NMP was used to exclude the signal in the Scatter gate. A stained sample with DiOC_2_(3) was used to assess the bacterial membrane potential using the 488 nm blue light laser using the Texas red filter (LP 670 nm) and the green filter (BP 530/30 nm). The ionophore CCCP and the chemosensitizer NMP caused a disruption/blockage on the membrane potential of *K. pneumoniae* resulting in a shift in the fluorescence.

FIGURE S2 Confirmation of the reproducibility of the RNA-seq data calculated as Spearman correlation coefficients from the normalized read counts obtained from two biological replicates.

TABLE S1; WS 1 Metabolic analysis – phenotypic Microarray analysis results on the NMP-mediated variations in *K. pneumoniae* metabolism.

TABLE S1; WS 2 RNA-seq metadata – details of the reads mapping against the genome of *K. pneumoniae* MGH 78578.

TABLE S1; WS 3 NMP RNA-seq – NMP based gene expression profiling of *K. pneumoniae* MGH 78578 during exposure to NMP.

TABLE S1; WS 4 qRT-PCR primers – sequences of qRT-PCR primers used in this study.
