# Supplementary figures and images for: Exposure to Sub-inhibitory Concentrations of the Chemosensitizer 1-(1-Naphthylmethyl)-Piperazine Creates Membrane Destabilization in Multi-Drug Resistant Klebsiella pneumoniae
Source: Front Microbiol. 2019 Feb 13;10:92. doi: 10.3389/fmicb.2019.00092 (PMC6381021; doi:10.3389/fmicb.2019.00092)

PBS + NMP 250  $\mu\text{g/mL}$

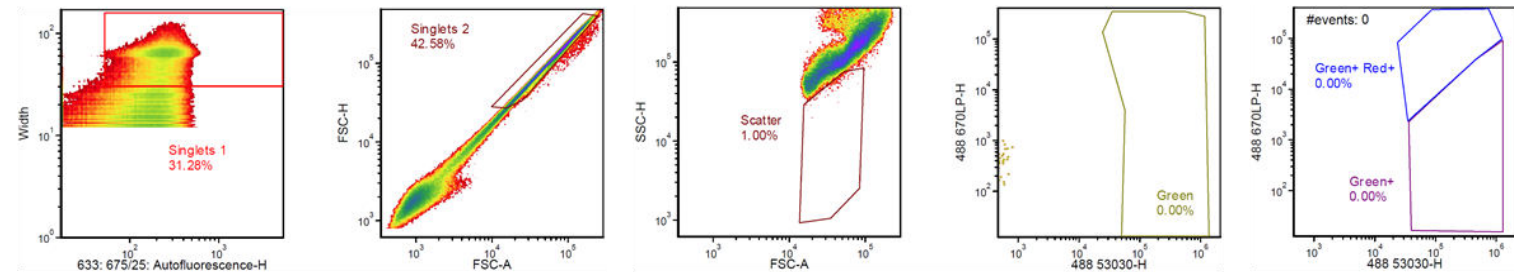

Cells + DiOC<sub>2</sub>(3)

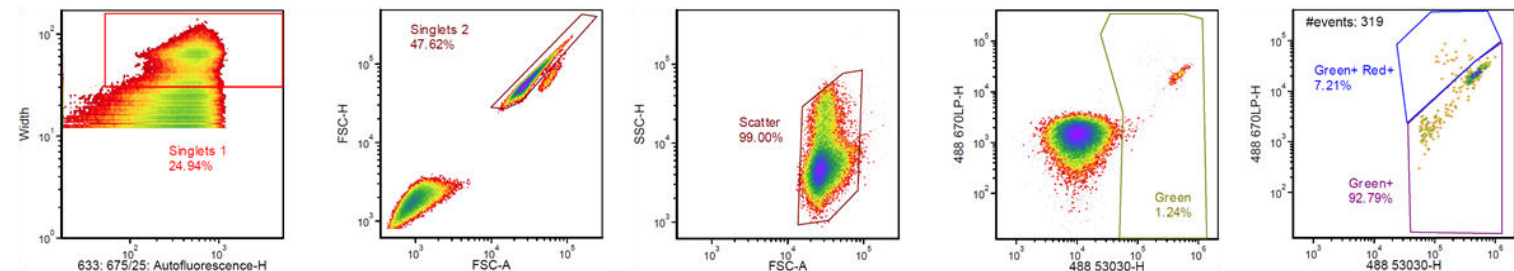

Cells + CCCP 50 mM  
+ DiOC<sub>2</sub>(3)

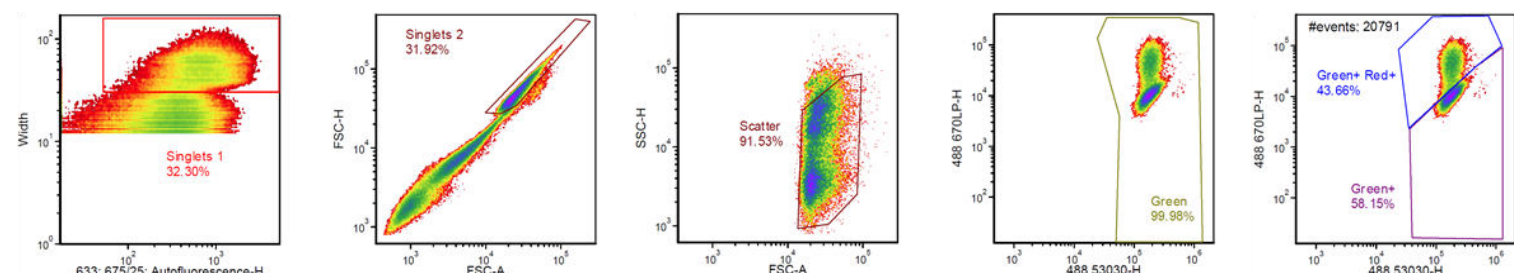

Cells + NMP 250  $\mu\text{g/mL}$  +  
DiOC<sub>2</sub>(3)

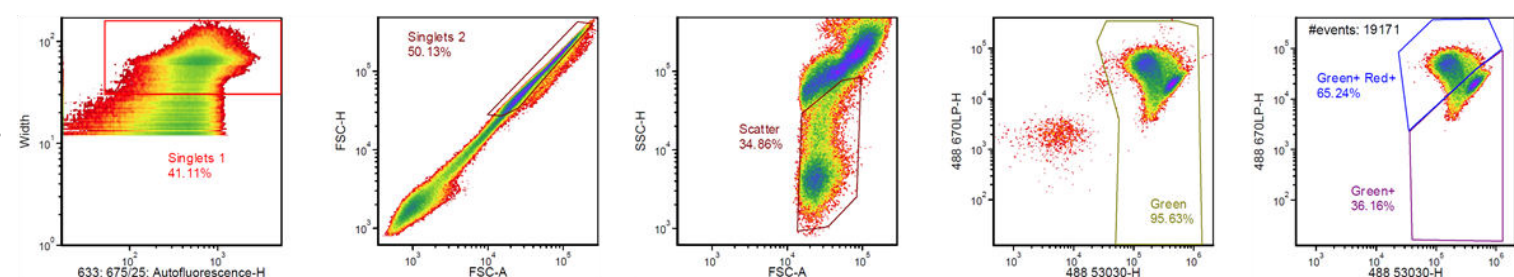

Supplement: FIGURE S1 — Sequential gating strategy applied to characterize K. pneumoniae MGH 78578 membrane potential shifts in the presence of the chemosensitizer NMP. The bacterial population was first identified with an unstained sample to check for autofluorescence using 633 nm laser with the filter 675/25 nm; gated samples (singlets 1) were next analyzed based on the forward scatter and gated as singlets 2. Finally, bacterial population was gated using the side scatter versus forward scatter. In order to ensure that the signal from the compounds did not affect the gating, a sample of NMP was used to exclude the signal in the Scatter gate. A stained sample with DiOC2(3) was used to assess the bacterial membrane potential using the 488 nm blue light laser using the Texas red filter (LP 670 nm) and the green filter (BP 530/30 nm). The ionophore CCCP and the chemosensitizer NMP caused a disruption/blockage on the membrane potential of K. pneumoniae resulting in a shift in the fluorescence. [file Data_Sheet_1.PDF]

# Comparison of biological replicates

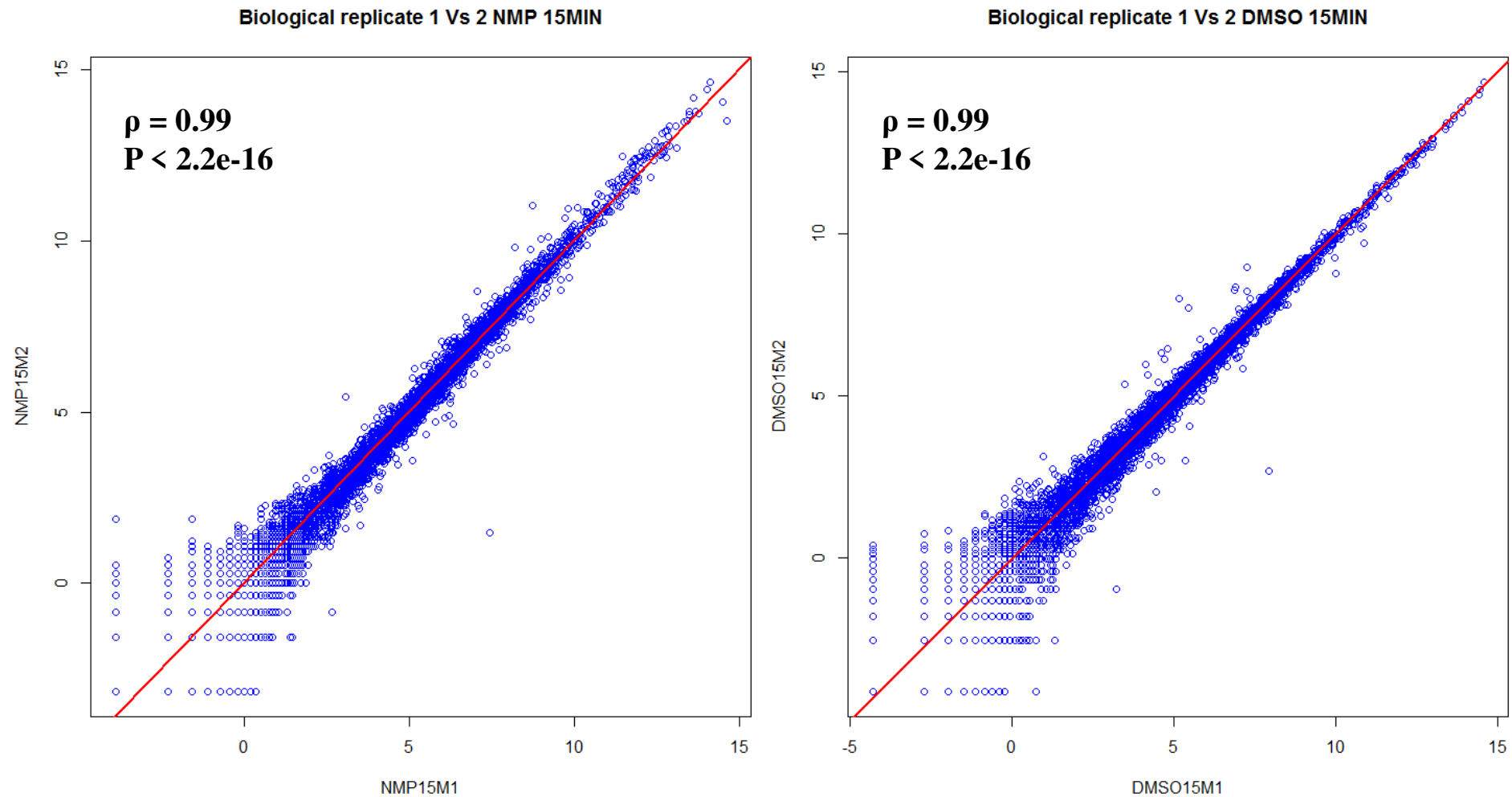

# Comparison of biological replicates

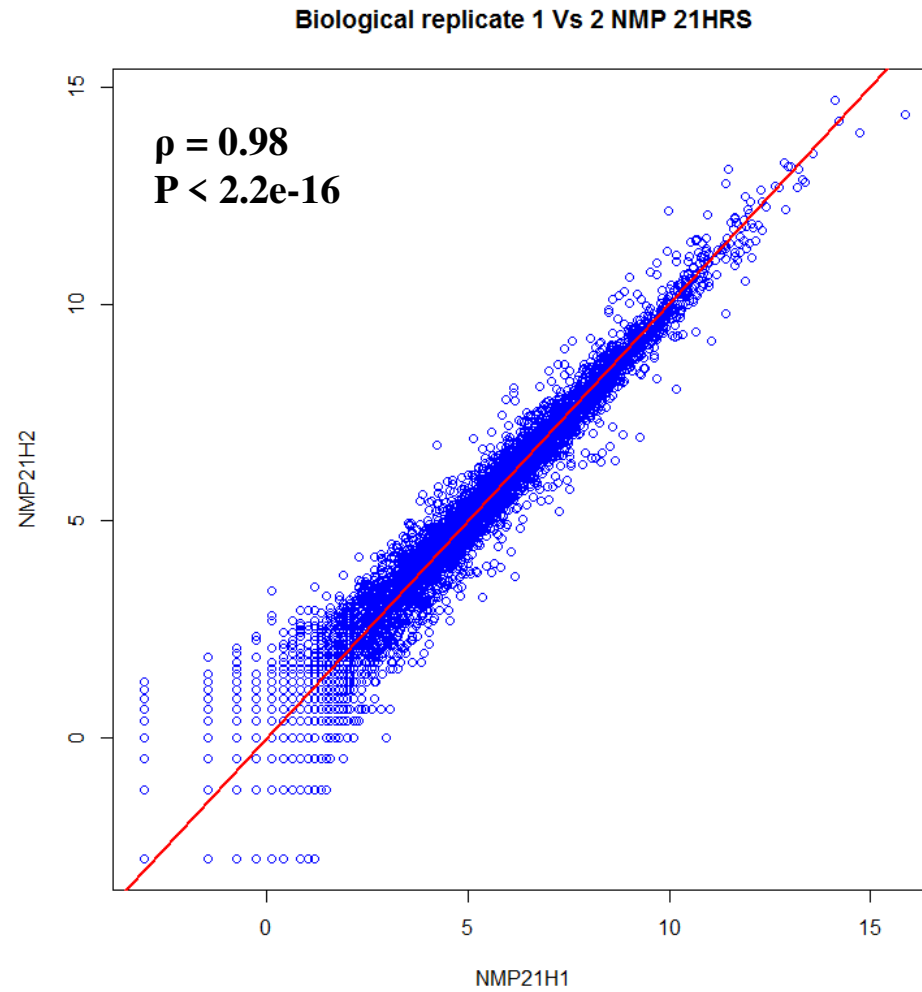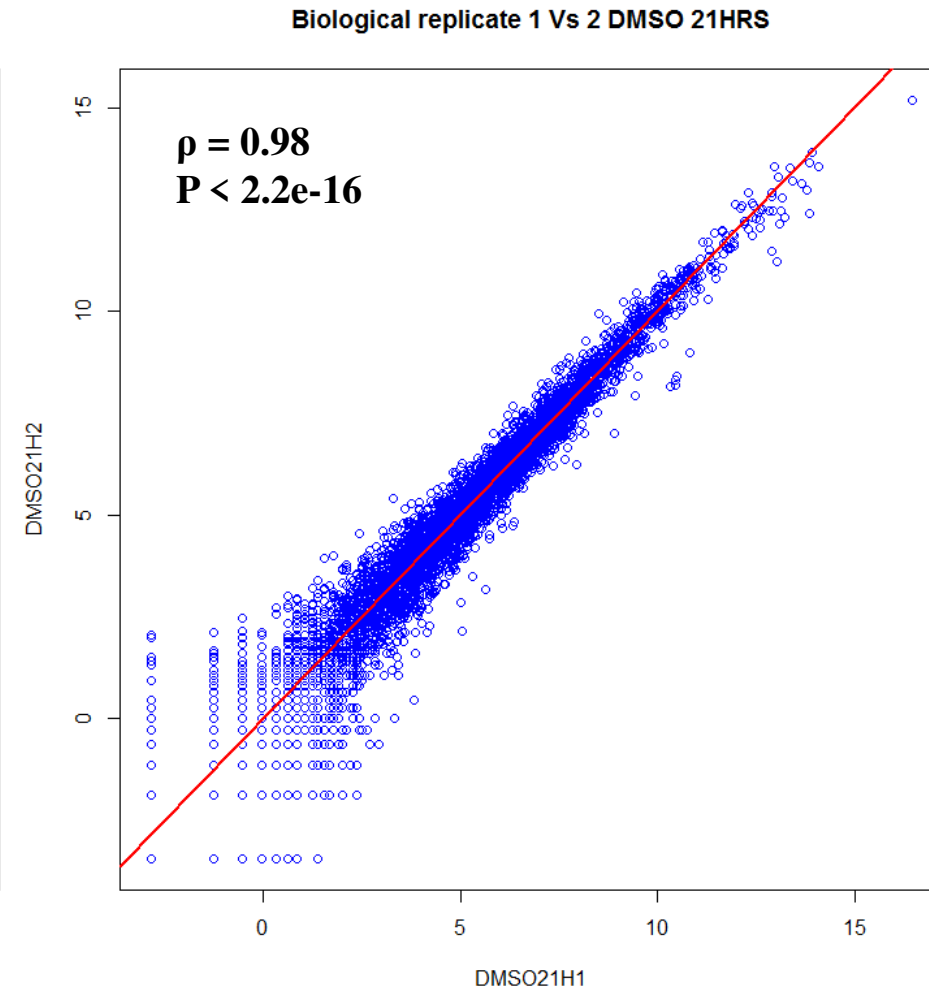

Supplement: FIGURE S2 — Confirmation of the reproducibility of the RNA-seq data calculated as Spearman correlation coefficients from the normalized read counts obtained from two biological replicates. [file Data_Sheet_2.PDF]
